# 320 mm InterTan nail optimizes biomechanics in AO/OTA 31A2.3 fractures: superior stress distribution, micromotion, and strain for enhanced healing

**DOI:** 10.3389/fbioe.2026.1677273

**Published:** 2026-02-02

**Authors:** Pao Wang, Shengjie Gu, Zhiwei Liu, Ning Li, Chengsong Lan, Biao Zhang, Gang Liu

**Affiliations:** 1 Emergency Department, The affiliated Hospital of Guizhou Medical University, Guiyang, Guizhou, China; 2 People’s Hospital of Dejiang, Department of Orthopedics, Tongren, Guizhou, China; 3 Xingyi City People’s Hospital, Department of Orthopedics, Xingyi, Guizhou, China; 4 Tongren City People’s Hospital, Department of Orthopedics, Tongren, Guizhou, China

**Keywords:** AO/OTA 31A2.3 fracture, biomechanics, fracture healing, InterTAN, long and short nail fixation

## Abstract

**Purpose:**

This study compares the biomechanical performance of InterTan nails of three lengths (180 mm, 240 mm, and 320 mm) in treating AO/OTA 31A2.3 comminuted intertrochanteric fractures, which are highly unstable and prone to fixation failure. The research question focuses on identifying the nail length that optimizes stress distribution, displacement, and strain to enhance fracture healing and reduce failure risk, thereby providing a theoretical foundation for clinical decision-making.

**Methods:**

Femoral CT images from a healthy 24-year-old male were used to reconstruct cortical and cancellous bone models in Mimics Research 21.0 and Geomagic Wrap 2021. A complete femur and AO/OTA 31A2.3 fracture model were constructed in SolidWorks 2022. InterTan models (180 mm, 240 mm, and 320 mm) were assembled with the fracture model, and finite element analysis (FEA) was performed in Ansys Workbench 18.0 under three loading conditions (standing, walking, and stair descent) to evaluate stress, deformation, and failure risk.

**Results:**

Stress concentrated at the nail-screw junction and proximal aperture, with the 180 mm nail exhibiting the highest stress, the 320 mm nail the lowest, and the 240 mm nail intermediate values. Displacement increased with nail length under dynamic loads, whereas the 180 mm nail minimized displacement during standing. The 240 mm nail showed the lowest strain during standing but the highest during stair descent. Differences in stress and displacement were statistically significant (P < 0.05).

**Conclusion:**

The 320 mm nail optimizes stress distribution, micromotion, and strain, thereby reducing failure risk and promoting healing. These findings align with biological osteosynthesis principles and support personalized treatment strategies.

## 1 Introduction

Proximal femoral fractures are among the most common orthopedic conditions, with incidence rates showing a strong positive correlation with age. Epidemiological data indicate that older adults, particularly those over 65 years, represent the highest-risk group, with a predominance among females. This elevated risk in women is closely linked to accelerated bone loss following postmenopausal estrogen decline. In contrast, among younger patients, males account for 89.9% of cases, with an annual incidence of 3.12 per 100,000. Primary injury mechanisms include high-energy trauma, such as falls from heights (48.1%) and traffic accidents (26.9%) (Maluta et al., 2019; Hantouly et al., 2024). In elderly patients, intertrochanteric fractures (36.1%) and femoral neck fractures (33.7%) are the most prevalent types (Ridzwan et al., 2018). Studies have demonstrated that reduced bone mineral density (BMD) significantly increases fracture risk, and BMD in patients with intertrochanteric fractures is typically lower than in those with femoral neck fractures (Ishizu et al., 2024; Maluta et al., 2019). Additional risk factors include COVID-19 infection, preoperative comorbidities (e.g., upper limb fractures), and hip geometric abnormalities (e.g., acetabular retroversion), which may elevate mortality or internal fixation failure rates (Becker et al., 2022; Joosse et al., 2019). Notably, mortality rates for proximal fractures (25%) exceed those for distal fractures (8.3%), with comorbidities contributing more to mortality in patients with normal BMD (Baghdadi et al., 2023; Joosse et al., 2019). Given the high disability rates and substantial socioeconomic burden, proximal femoral fractures pose a major public health challenge in aging societies (Maluta et al., 2019; Silva et al., 2018).

According to the AO/OTA classification system, intertrochanteric fractures are categorized as subtype 31A2, with stability decreasing from 31A2.1–31A2.3. Biomechanical studies reveal that the strength of type 31A2.3 fractures is approximately 40% lower than that of type 31A2.1 (Ling et al., 2022). Clinical observations indicate that type 31A2.3 accounts for 50.7% of unstable fractures, often presenting as four-part fractures (Jensen type 5) (Verzellotti et al., 2025). The classification features are as follows: Type 31A2.1 involves a two-part fracture of the greater trochanter, with the lesser trochanter intact or minimally avulsed, offering relatively high stability. Type 31A2.2 is a three-part fracture separating the greater and lesser trochanters, classified as moderately stable. Type 31A2.3 is a multiplanar comminuted fracture (≥5 fragments) involving the medial/posterior cortex (lesser trochanter), representing an extremely unstable variant. Treatment goals emphasize anatomic reduction, stable fixation, and functional recovery, with reconstruction of medial cortical support being critical (Ling et al., 2022; Verzellotti et al., 2025). Currently, open reduction and internal fixation are the primary approaches. For unstable fractures like type 31A2.3, intramedullary fixation systems (e.g., PFNA, InterTan) are preferred (Fan et al., 2025; Luo et al., 2020; Warschawski et al., 2022). However, these procedures demand high surgical expertise, and elderly patients face elevated risks of postoperative complications, including nonunion, fixation failure, and infection (Hao et al., 2019; Tomás-Hernández et al., 2018). In high-energy trauma cases, deep infection rates (2%) and reoperation rates (7%) are notably higher (Fu et al., 2020; Klopfer et al., 2018).

Finite element analysis (FEA) originated in the 1940s, initially developed by mathematicians and engineers in aerospace to address complex structural mechanics problems. Its theoretical foundation was proposed by Richard Courant in 1943 (Courant, 1943). In the 1950s, advancements in computer technology enabled FEA implementation, leading to widespread engineering applications (Huiskes and Chao, 1983). By the 1960s, FEA extended to civil engineering, and post-1970s improvements in computational power facilitated its expansion into biomedicine. In orthopedics, FEA was applied to bone mechanics in the 1980s, simulating stress distribution and deformation in bones, joints, and implants (Viceconti et al., 2005). Contemporary orthopedic FEA supports artificial joint design (Ky et al., 2025), fracture healing evaluation (Yi et al., 2025), implant optimization (Yidan et al., 2025), and prediction of mechanical behavior in conditions like osteoporosis (Shuyu et al., 2025). By accurately modeling complex biological structures, FEA advances personalized medicine (Mallinos and Jones, 2024) and surgical planning (Mi, 2025).

InterTan nail lengths range from 180 mm to 460 mm, while most Chinese adult femurs measure approximately 38–42 cm. No consensus exists on the optimal nail length for AO/OTA 31A2.3 fractures. This FEA study compares stress concentration, failure risk, fracture gap displacement, and strain absorption for 180 mm, 240 mm, and 320 mm InterTan nails under standing, slow walking, and stair descent conditions. The aim is to address the lack of theoretical guidance in nail length selection and provide evidence-based recommendations for treating AO/OTA 31A2.3 fractures.

## 2 Materials and methods

### 2.1 Materials

A healthy 24-year-old male volunteer (weight: 60 kg, height: 170 cm, no history of femoral trauma or disease) underwent digital radiography (DR) at The Affiliated Hospital of Guizhou Medical University to exclude pathology or trauma. Spiral CT imaging of the right hip and femur was performed using a Siemens 64-slice scanner. The volunteer was positioned supine, with the scanning area centered using a calibration phantom. Parameters included 120 kV, 125 mA, and 0.625 mm slice thickness, capturing continuous axial images from the hip to the femur. Raw data were interpolated, magnified, saved in DICOM format, and stored on a CD. The study received ethics approval from the hospital’s committee (Approval No. 2304021), and informed consent was obtained.

### 2.2 Methods

#### 2.2.1 Three-dimensional reconstruction of the intact femur

**FIGURE 1:**
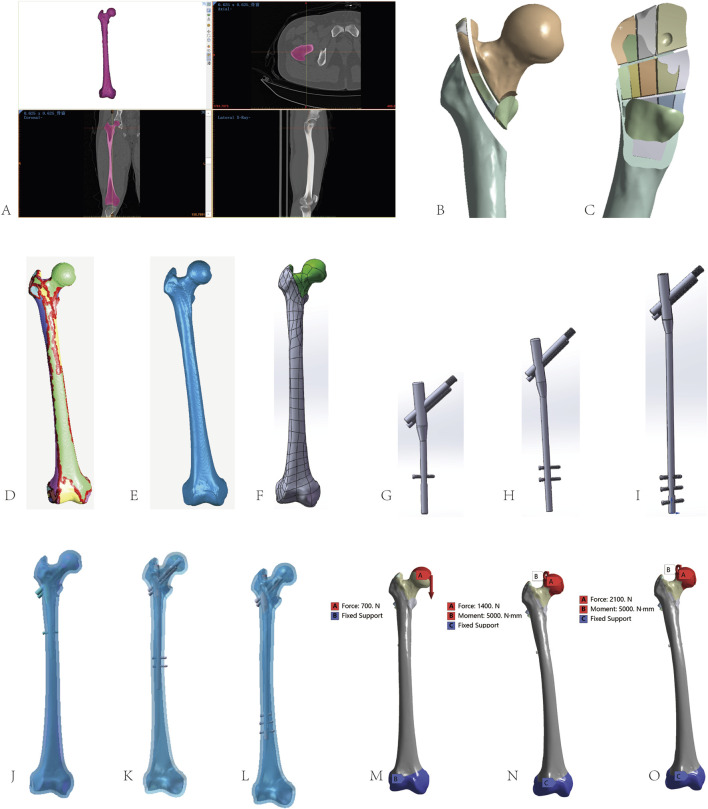
Construction of the complete femoral model: **(A)** 3D femoral image extracted in Mimics. **(B,C)** Schematic diagrams of fracture line construction in SolidWorks. **(D–F)** Femoral geometry constructed in Geomagic. **(G)** InterTan model with a 180-mm main nail generated in SolidWorks. **(H)** InterTan model with a 240-mm main nail generated in SolidWorks. **(I)** InterTan model with a 320-mm main nail generated in SolidWorks. **(J)** Constraint-based assembly simulation of the 180-mm InterTan implant and fractured femur in SolidWorks Motion Analysis. **(K)** Constraint-based assembly simulation of the 240-mm InterTan implant and fractured femur in SolidWorks Motion Analysis. **(L)** Constraint-based assembly simulation of the 320-mm InterTan implant and fractured femur in SolidWorks Motion Analysis. **(M)** Schematic of 700 N gravitational loading on the femoral head and distal fixation position. **(N)** Schematic of 1400 N gravitational loading on the femoral head, 5 N m torque direction, and distal fixation position. **(O)** Schematic of 2100 N gravitational loading on the femoral head, 5 N m torque direction, and distal fixation position.

Bone and soft tissue boundaries were delineated using optimal thresholding in Mimics Research 21.0. Soft tissues were removed via Threshold, Region Grow, Split Mask, Edit Mask, and Multiple Slice Edit tools to isolate the femoral contour. Gaps were filled using Cavity Fill and Smart Fill, and the model was smoothed to satisfaction. A 3D femoral model was generated using Calculate Part (Figure 1A) and exported as a binary STL file. In Geomagic Wrap 2021, Mesh Doctor analyzed and repaired polygonal meshes, resolving non-manifold edges, self-intersections, highly creased edges, spikes, small components, tunnels, and holes (Figure 1D). Retriangulation, spike removal, sanding, filling, and smoothing eliminated non-characteristic defects, with sharp edges smoothed to avoid high-curvature self-intersections. Smooth surface patches were fitted in the Exact Surface module. Cancellous bone was created by offsetting the cortical bone’s inner surface based on CT-derived thickness, yielding a complete femoral model (Figure 1E). The model was saved as an STL file, imported into SolidWorks 2022, aligned to the origin, and assembled to produce cortical and cancellous bone components, verified for interference (Figure 1F).

#### 2.2.2 Fracture model construction

In SolidWorks 2022, the right femur was oriented in the coronal plane, and a fracture sketch was drawn based on a schematic (Figure 1B). Cortical and cancellous bone were segmented, adjusted to the sagittal plane, and a comminuted fracture sketch was created to form an AO/OTA 31A2.3 model with intertrochanteric fragments (Figure 1C).

#### 2.2.3 InterTan model construction

Using Tri-Max InterTan parameters from Smith & Nephew (main nail: φ9.0 mm × 180–320 mm; distal locking screw: φ5 mm × 45 mm; compression screw: φ10 mm × 90 mm; lag screw: φ7 mm × 85 mm), InterTan models were built in SolidWorks 2022 (Figures 1G–I) and exported as STP files.

#### 2.2.4 Finite element model construction

In SolidWorks 2022, the AO/OTA 31A2.3 fracture model and three InterTan models (180 mm, 240 mm, 320 mm) were assembled, with combination cuts and Boolean operations creating three FEA models (Figures 1J–L), exported as X_T files. These were imported into Ansys Workbench 18.0’s Static Structural module for assignment of boundary conditions, loads, and material properties, followed by meshing and analysis.

### 2.3 Material properties and meshing

Cortical and cancellous bone were modeled as isotropic, homogeneous linear elastic materials, incorporating nonlinear effects and thermal strain (Goosen et al., 2009). Properties included: cortical bone (elastic modulus: 16,800 MPa, Poisson’s ratio: 0.29); cancellous bone (elastic modulus: 840 MPa, Poisson’s ratio: 0.2); InterTan (TC4 titanium alloy, elastic modulus: 110,000 MPa, Poisson’s ratio: 0.30) (Wang et al., 2025). Models were meshed with Solid45 elements (size: 2.0 mm), validated by convergence tests (stress change <3%, mesh size change <5%). Element types, counts, and node counts are listed in Table 1.

**TABLE 1:** Mesh details for model components.

Component	Element type	Element count	Node count
Cortical bone	Solid45	382,878	580,018
Cancellous bone	Solid45	261,615	381,188
180 mm InterTan	Solid45	14,417	8,558
240 mm InterTan	Solid45	161,654	253,982
320 mm InterTan	Solid45	182,891	286,402

### 2.4 Boundary conditions and loads

Component friction was modeled as surface-to-surface contact (tolerance: 0.1), with coefficients of 0.46 for fracture surfaces and 0.42 for bone-implant interfaces; cortical-cancellous bone and nail-screw interfaces were bonded (Goffin et al., 2013). Femoral loads are complex, with hip joint forces ranging from 2.6 to 4.1 times body weight during normal activities. Muscle forces were simplified (Taylor et al., 1996). Applied loads were 700 N for standing, 1400 N for slow walking, and 2100 N for stair descent, with a 5 N m torque during walking and stair descent to simulate rotation (Taylor et al., 1996). Distal femoral condyles were fully constrained (Figures 1M–O).

### 2.5 Model validation

Most FEA studies validate models using metrics consistent with prior research. To confirm the reliability of our intact femoral model, we referenced established methods and compared it with biomechanical cadaveric studies (Wang et al., 2025; Zhou et al., 2024). This comparison establishes the model’s credibility for subsequent analyses.

### 2.6 Outcome measures

The top 10 integration points from FEA results were evaluated for femoral displacement, fracture gap displacement, femoral strain, and von Mises stress under three gait conditions.

### 2.7 Statistical analysis

Von Mises stress, maximum model and fracture gap displacement, and overall strain were computed to assess stress distribution and mechanical stability. Higher von Mises stress indicates increased local concentration and implant failure risk. Greater displacement suggests enhanced fracture mobility, promoting healing via micromotion. The 180 mm nail served as the control due to its common use. Percentage difference (PD) was calculated as: PD = (Pa - P1)/P1 × 100%, where Pa denotes values for 240 mm or 320 mm nails, and P1 for the 180 mm nail. Data were analyzed using SPSS 22.0 (IBM, United States). Normally distributed data with equal variances underwent paired t-tests; non-normal data were assessed with Kruskal–Wallis one-way ANOVA. Significance was set at α = 0.05 (P < 0.05).

## 3 Results

### 3.1 Model validation

**FIGURE 2:**
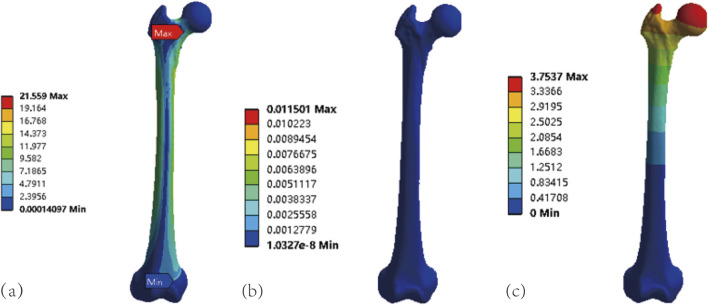
Validation of the femoral model: **(a)** Stress cloud diagram of the intact femoral model under 700 N load. **(b)** Strain cloud diagram of the intact femoral model under 700 N load. **(c)** Displacement cloud diagram of the intact femoral model under 700 N load.

A 700 N load was applied to the femur, and stress and strain cloud diagrams were observed (Figure 2). Results showed maximum stress and strain concentrations similar to prior studies. Maximum von Mises stress and stresses at eight points on the AO/OTA 31A2.3 fracture cross-section were compared with previous FEA and cadaveric studies (Tables 2, 3). Our intact femur’s maximum von Mises stress was 21.559 MPa, slightly higher than 17.49–18.05 MPa reported by San Antonio et al. (2012). Stresses at eight femoral neck cross-section points exceeded those in Jian-Qiao Peng et al. (2020) and Zhou et al. (2024) models but fell within ranges from Zhang et al. (2009). These findings confirm the model’s suitability for further investigation.

**TABLE 2:** The maximum von-Mises stress of the femoral model.

Compared studies	San (1) 2012	San (2) 2012	San (3) 2012	Zhou 2024	Own
Outcomes	17.95	17.49	18.05	17.649	21.559

**TABLE 3:** Maximum von-mises stresses at selected points on the Femoral Pertrochanteric cross-section of the FE Model.

Compared studies		The maximum von-mises stresses at 8 points on the femoral neck cross-section (MPa)							
		A	B	C	D	E	F	G	H
Matthew 2020	FE model	1.7486	1.0082	1.5609	0.476	2.9542	2.0889	1.1033	0.6937
	Cadaver	2.0805	0.8395	2.4638	0.2555	3.2609	2.7923	1.3688	0.219
Zhang G 2009	FE model 1	5.6195	2.754	2.7657	2.7075	2.7044	2.6721	2.6699	5.6195
	FE model 2	4.6121	2.5308	2.524	2.6403	2.6073	2.6197	2.6159	4.6121
	FE model 3	13.4386	6.6241	6.5349	6.9777	6.974	6.8769	6.8681	13.4386
	FE model 4	22.9186	8.1142	8.3652	8.3234	8.3628	8.3917	8.3884	22.9186
Zhou 2025		6.6864	2.5484	2.5166	3.5549	7.3338	6.0198	2.2854	4.2151
Own		14.613	5.2354	5.1803	6.124	9.1149	13.4634	6.9704	12.0973

### 3.2 Stress distribution in InterTan models

**FIGURE 3:**
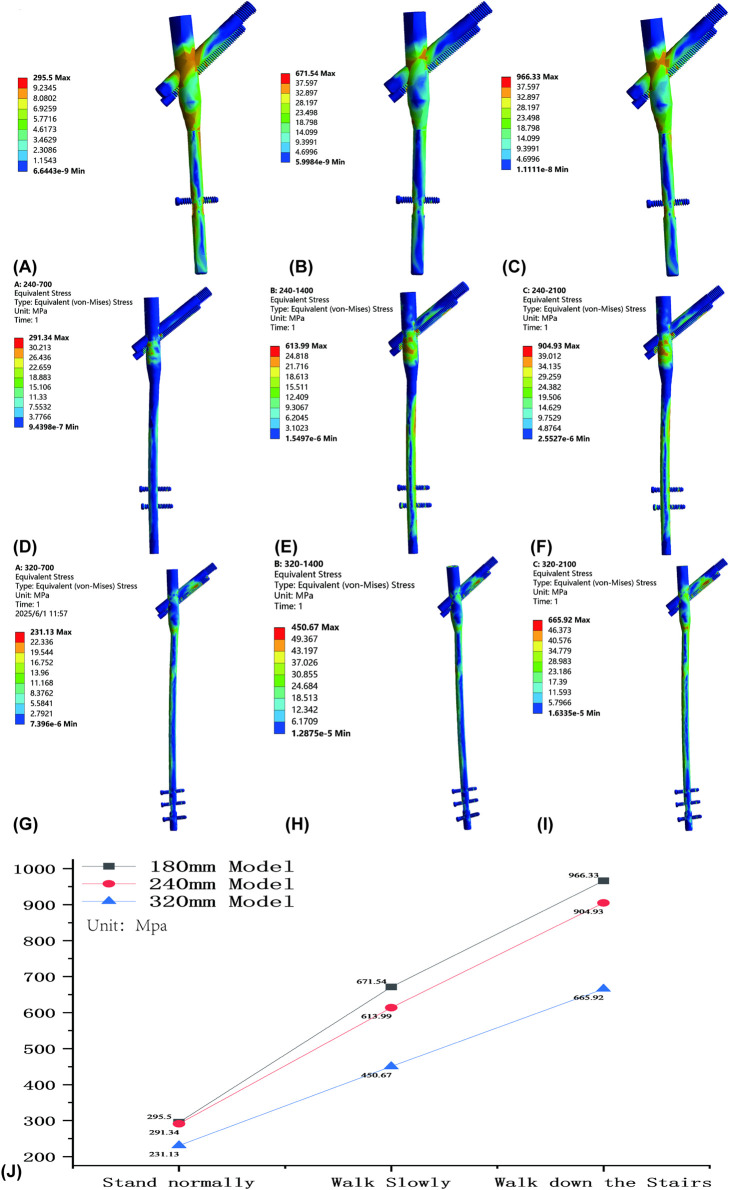
Stress cloud diagrams of AO/OTA 31A2.3 fracture models fixed with varying InterTan lengths under different gaits: **(A)** 180-mm InterTan during standing. **(B)** 180-mm InterTan during walking. **(C)** 180-mm InterTan during stair descent. **(D)** 240-mm InterTan during standing. **(E)** 240-mm InterTan during walking. **(F)** 240-mm InterTan during stair descent. **(G)** 320-mm InterTan during standing. **(H)** 320-mm InterTan during walking. **(I)** 320-mm InterTan during stair descent. **(J)** Stress line charts for varying InterTan lengths under different gaits.

Under three gait conditions, stress concentrated at the main nail-screw junction and proximal aperture, aligning with clinical failure sites (Figures 3A–I). Stress decreased with increasing nail length: highest for 180 mm, intermediate for 240 mm, and lowest for 320 mm. For standing, stresses were 214.53 ± 36.78 MPa (180 mm), 206.52 ± 41.21 MPa (240 mm), and 176.38 ± 31.76 MPa (320 mm). For walking, values were 477.42 ± 88.48 MPa (180 mm), 440.81 ± 84.33 MPa (240 mm), and 359.76 ± 58.06 MPa (320 mm). For stair descent, stresses were 691.03 ± 125.23 MPa (180 mm), 647.19 ± 125.41 MPa (240 mm), and 534.93 ± 89.24 MPa (320 mm) (Table 4). Stress intensified with activity level, with significant differences between lengths (P < 0.05, Table 5 and Figure 3J).

**TABLE 4:** Von mises stress and statistical comparisons across gait conditions.

Nail length	Standing (MPa)	Walking (MPa)	Stair descent (MPa)
180 mm	214.53 ± 36.79	477.42 ± 88.85	691.03 ± 125.24
240 mm	206.53 ± 41.22	440.82 ± 84.33	647.19 ± 125.42
320 mm	176.39 ± 31.77	359.76 ± 58.07	534.93 ± 89.25

Data represent mean ± SD (n = 10 integration points).

**TABLE 5:** Paired t-test results among different lengths of InterTan for treating AO 31A2.3 fractures.

Gait	Pair name	Pair 1 (P1, mean ± SD)	Pair 2 (P2, mean ± SD)	Difference (P1-P2)	t-value	p-value
Normal standing	180 VS 240 mm	214.53 ± 36.79	206.53 ± 41.22	8.00	2.092	0.066
	180 VS 320 mm	214.53 ± 36.79	176.39 ± 31.77	38.14	8.728	0.000^**^
	240 VS 320 mm	206.53 ± 41.22	176.39 ± 31.77	30.14	5.049	0.001^**^
Slow walking	180 VS 240 mm	477.42 ± 88.85	440.82 ± 84.33	36.61	5.523	0.000^**^
	180 VS 320 mm	477.42 ± 88.85	359.76 ± 58.07	117.66	8.862	0.000^**^
	240 VS 320 mm	440.82 ± 84.33	359.76 ± 58.07	81.05	6.901	0.000^**^
Stair descent	180 VS 240 mm	691.03 ± 125.24	647.19 ± 125.42	43.84	4.608	0.001^**^
	180 VS 320 mm	691.03 ± 125.24	534.93 ± 89.25	156.10	8.518	0.000^**^
	240 VS 320 mm	647.19 ± 125.42	534.93 ± 89.25	112.26	5.818	0.000^**^

^a^ p < 0.05, **p < 0.01.

### 3.3 Femoral displacement

**FIGURE 4:**
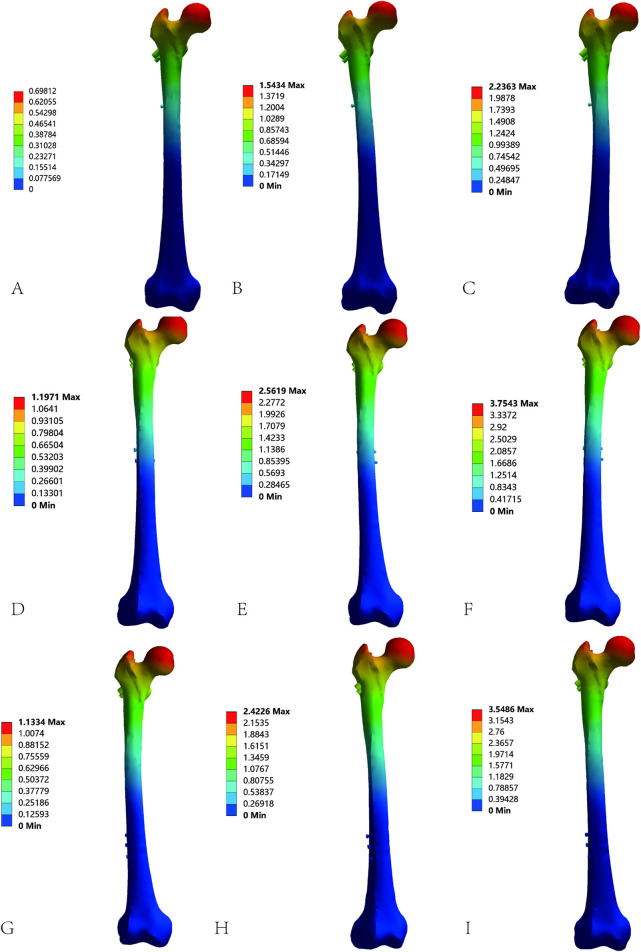
Total displacement cloud diagrams of AO/OTA 31A2.3 fracture models fixed with varying InterTan lengths under different gaits: **(A)** 180-mm InterTan during standing. **(B)** 180-mm InterTan during walking. **(C)** 180-mm InterTan during stair descent. **(D)** 240-mm InterTan during standing. **(E)** 240-mm InterTan during walking. **(F)** 240-mm InterTan during stair descent. **(G)** 320-mm InterTan during standing. **(H)** 320-mm InterTan during walking. **(I)** 320-mm InterTan during stair descent.

Femoral displacement varied by nail length and gait (Figure 4; Table 6). During standing, the 180 mm nail showed minimal displacement (0.693 ± 0.004 mm), while 240 mm (1.128 ± 0.000 mm) and 320 mm (1.129 ± 0.003 mm) were comparable. For walking, displacements increased to 1.532 ± 0.008 mm (180 mm), 2.409 ± 0.001 mm (240 mm), and 2.263 ± 0.001 mm (320 mm), with longer nails exhibiting greater displacement under dynamic loads. For stair descent, values were 2.219 ± 0.011 mm (180 mm), 3.739 ± 0.011 mm (240 mm), and 3.316 ± 0.001 mm (320 mm), confirming a positive correlation between nail length and displacement.

**TABLE 6:** Maximum femoral displacement across gait conditions.

Nail length	Standing (mm)	Walking (mm)	Stair descent (mm)
180 mm	0.693 ± 0.004	1.532 ± 0.008	2.219 ± 0.011
240 mm	1.128 ± 0.000	2.409 ± 0.001	3.739 ± 0.011
320 mm	1.129 ± 0.003	2.263 ± 0.001	3.316 ± 0.001

Data represent mean ± SD (n = 10 integration points).

### 3.4 Fracture gap displacement

**FIGURE 5:**
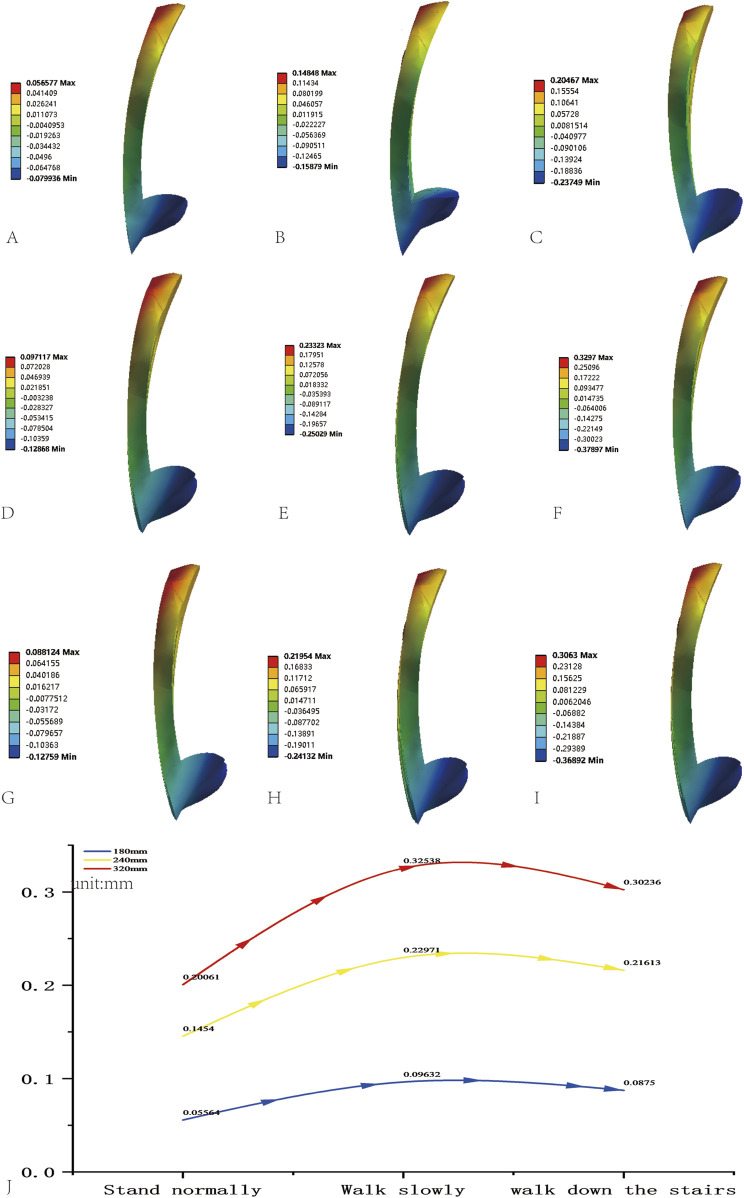
Fracture fragment displacement cloud diagrams in AO/OTA 31A2.3 models fixed with varying InterTan lengths under different gaits: **(A)** 180-mm InterTan during standing. **(B)** 180-mm InterTan during walking. **(C)** 180-mm InterTan during stair descent. **(D)** 240-mm InterTan during standing. **(E)** 240-mm InterTan during walking. **(F)** 240-mm InterTan during stair descent. **(G)** 320-mm InterTan during standing. **(H)** 320-mm InterTan during walking. **(I)** 320-mm InterTan during stair descent. **(J)** Fracture fragment displacement line charts for varying InterTan lengths under different gaits.

Fracture gap displacement increased with nail length across conditions (Figures 5A–I; Table 7), showing a positive linear correlation (Figure 5J). Differences were significant (P < 0.05, Table 8). For standing (700 N), displacements were 0.0556 ± 0.0007 mm (180 mm), 0.1454 ± 0.0019 mm (240 mm, 1.6-fold higher), and 0.2006 ± 0.0027 mm (320 mm, 2.61-fold higher). Short nails limited micromotion, while longer nails enhanced it via elastic deformation. For walking (1400N + 5 N m), values were 0.0963 ± 0.0007 mm (180 mm), 0.2297 ± 0.0022 mm (240 mm), and 0.3254 ± 0.0029 mm (320 mm), with a 62.2% increase for 320 mm. For stair descent (2100N + 5 N m), displacements were 0.0875 ± 0.0005 mm (180 mm), 0.2161 ± 0.0021 mm (240 mm), and 0.3024 ± 0.0027 mm (320 mm). The 240 mm nail showed no significant change from walking (P = 0.07), while the 320 mm maintained an upward trend.

**TABLE 7:** Fracture gap micromotion across gait conditions.

Nail length	Standing (mm)	Walking (mm)	Stair descent (mm)
180 mm	0.056 ± 0.001	0.096 ± 0.001	0.088 ± 0.001
240 mm	0.145 ± 0.002	0.230 ± 0.002	0.216 ± 0.002
320 mm	0.201 ± 0.003	0.325 ± 0.003	0.302 ± 0.003

**TABLE 8:** Pairwise t-test results for fracture gap micromotion.

Gait	Comparison	Mean difference (mm)	t-value	P-value
Standing	180 vs. 240 mm	−0.090	−220.70	0.000**
​	180 vs. 320 mm	−0.145	−223.73	0.000**
​	240 vs. 320 mm	−0.055	−206.21	0.000**
Walking	180 vs. 240 mm	−0.133	−272.02	0.000**
​	180 vs. 320 mm	−0.229	−319.91	0.000**
​	240 vs. 320 mm	−0.096	−314.674	0.000**
Stair descent	180 vs. 240 mm	−0.129	−253.54	0.000**
​	180 vs. 320 mm	−0.215	−305.69	0.000**
​	240 vs. 320 mm	−0.086	−306.31	0.000**

Data represent mean ± SD (n = 10 integration points). **P < 0.01.

### 3.5 Femoral strain

**FIGURE 6:**
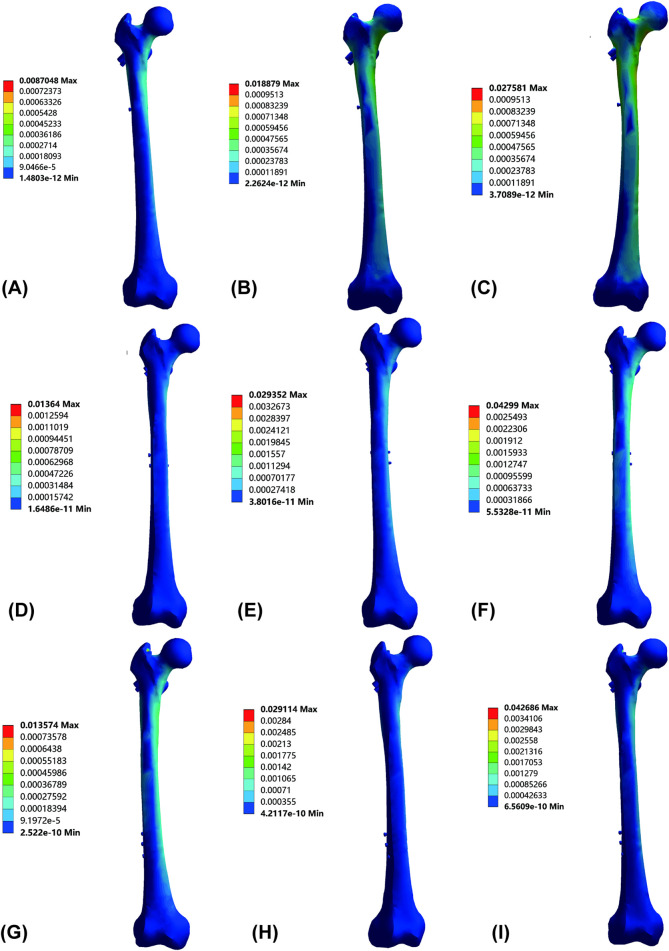
Strain cloud diagrams of AO/OTA 31A2.3 fracture models fixed with varying InterTan lengths under different gaits: **(A)** 180-mm InterTan during standing. **(B)** 180-mm InterTan during walking. **(C)** 180-mm InterTan during stair descent. **(D)** 240-mm InterTan during standing. **(E)** 240-mm InterTan during walking. **(F)** 240-mm InterTan during stair descent. **(G)** 320-mm InterTan during standing. **(H)** 320-mm InterTan during walking. **(I)** 320-mm InterTan during stair descent.

Strain varied by nail length and condition (Figure 6; Table 9). During standing, the 240 mm nail had the lowest strain (0.003 ± 0.000), followed by 180 mm (0.006 ± 0.001) and 320 mm (0.008 ± 0.002). For walking, strains were 0.013 ± 0.002 (180 mm), 0.005 ± 0.001 (240 mm), and 0.004 ± 0.001 (320 mm), with longer nails providing better control. For stair descent, values were 0.019 ± 0.003 (180 mm), 0.026 ± 0.007 (240 mm), and 0.006 ± 0.001 (320 mm), highlighting the 320 mm nail’s superior performance. Nail length effects on strain were condition-specific, with short nails stable under static loads and 320 mm nails excelling under dynamic loads.

**TABLE 9:** Maximum femoral strain across gait conditions.

Nail length	Standing	Walking	Stair descent
180 mm	0.006 ± 0.001	0.013 ± 0.002	0.019 ± 0.003
240 mm	0.003 ± 0.000	0.005 ± 0.001	0.026 ± 0.007
320 mm	0.008 ± 0.002	0.004 ± 0.001	0.006 ± 0.001

## 4 Discussion

This FEA study evaluated the biomechanical effects of varying InterTan nail lengths on AO/OTA 31A2.3 fractures, demonstrating significant influences on failure risk, displacement, and strain, with key clinical implications.

### 4.1 Stress distribution and refracture risk

The 320 mm nail showed markedly lower von Mises stress (21.7%–29.8% reduction vs. 180 mm) across conditions, attributable to its extended lever arm dispersing stress over a broader bone-nail interface, mitigating concentration at the nail-screw junction (691.03 MPa for 180 mm). This concurs with clinical observations of InterTan failure locations (Lack et al., 2024). Research links stress concentration to fatigue failure; Daner Iii et al. (2017) reported higher concentrations with short nails, elevating risk, whereas long nails alleviate stress and refracture potential. Daner et al. noted short nails induce femoral shaft stress, averted by long nails. Je et al. (2024) found a 74.9% lower yield risk with ≥320 mm nails in osteoporotic subtrochanteric fractures. Our results affirm that 320 mm nails minimize failure and refracture by stress distribution, especially in active patients.

### 4.2 Micromotion and fracture healing

Short nails exhibited slightly lower micromotion (8% difference) under physiological loads, but long nails may amplify fracture gap displacement under dynamic loads (>800 N) due to distal elastic deformation (Udin et al., 2024). This aligns with our data. Prior studies indicate short nails may inadequately buffer impact forces, limiting stability via the “elastic deformation space” (unsupported bone) (Luo et al., 2020; Tang et al., 2024). Nail length must suit fracture type, as extremes compromise stability.

Local micromotion critically regulates healing: moderate levels (0.05–0.2 mm) foster callus formation, while excess (>0.5 mm) promotes fibrous tissue (Je et al., 2024; Lv et al., 2025; Tucker et al., 2019). Here, the 320 mm nail’s walking displacement (0.325 mm) fits the “effective micromotion range,” likely from enhanced elastic space. Conversely, the 180 mm nail’s rigid fixation (0.0556 mm) may hinder callus via insufficient motion. This supports Glatt et al. (2021) micromotion threshold, underscoring the balance between stability and motion for optimal healing.

### 4.3 Strain distribution and mechanical regulation of fracture healing

Strain effects varied by length and load. Under static conditions, the 240 mm nail minimized strain (0.003), possibly due to alignment with the femoral axis for uniform distribution. Under dynamic loads, the 320 mm nail resisted impact better, with 0.006 strain during stair descent vs. 0.026 for 240 mm, absorbing energy via a longer arm. Longer nails enhance stability by extending the lever arm; Cha et al. (2024) confirmed this in PFNA for intertrochanteric fractures, linking length to strain. Martínez-Aznar et al. (2023) showed titanium nails permit greater micromotion under dynamic locking. The 240 mm nail’s stair descent strain surge (0.026) may stem from a “stress transition zone,” lacking long-nail absorption or short-nail rigidity, mirroring PFNA strain patterns (Linhart et al., 2023).

From a healing viewpoint, strain signals osteoblast differentiation via matrix deformation. Wang et al. (2017) noted 1,000–3,000 με promotes intramembranous ossification, while >5,000 με inhibits it. The 320 mm nail’s dynamic strains (0.004–0.006) optimize osteogenesis, whereas the 240 mm’s stair descent strain (0.026) risks damage. This echoes Wang et al.'s emphasis on implant strain absorption influencing the fracture microenvironment. Kogure et al. (2019) showed appropriate motion boosts callus proliferation and mechanics. The 320 mm nail’s static strain (0.008) remains below tolerance, ensuring safety under physiological loads.

While our finite element results indicate biomechanical advantages of the 320 mm InterTan nail (reduced von Mises stress and favorable dynamic strain/micromotion profiles), these computational findings are strengthened when considered alongside recent clinical evidence. A retrospective case series from the Hospital for Traumatology and Orthopaedics reported favorable outcomes using the Trigen InterTan nail in a cohort of mostly elderly patients (mean age ≈71 years), with a high union rate (97.14%), low complication rates and acceptable operative metrics (operative time, blood loss, length of stay). This clinical report supports the feasibility and effectiveness of InterTan constructs in older patients with comorbidities and suggests that the biomechanical benefits of longer nails demonstrated here may translate into improved clinical performance in geriatric populations. Nevertheless, selection of nail length must remain individualized—accounting for patient femoral geometry, bone quality, fracture morphology, and surgeon preference—because clinical outcomes depend on surgical technique, reduction quality and patient factors that FEA cannot capture (Nguyen et al., 2024).

## 5 Limitations and future work

Limitations and future directions—Several limitations of this study warrant emphasis. First, the FEA was constructed from a single healthy 24-year-old male femur model, which does not capture the anatomical variability and reduced bone mineral density typical of the elderly patients who most commonly sustain AO/OTA 31A2.3 fractures. Bone quality, cortical thickness, femoral curvature and neck-shaft angle vary substantially with age, sex and ethnicity, and these factors will influence implant–bone load transfer and micromotion. Second, simplifications inherent to our modelling (linear elastic isotropic bone properties, simplified muscle loading, bonded interfaces at some contacts) limit direct extrapolation to the clinical setting. Third, clinical outcomes are materially affected by reduction quality, comorbidities and rehabilitation—factors outside the scope of computational modelling. To address these limitations we recommend future studies that (1) use a cohort of patient-specific models spanning age ranges and osteoporotic bone conditions, (2) perform sensitivity analyses on bone material properties and muscle loads, and (3) integrate computational results with prospective clinical datasets (for example, multi-center registries or targeted case series such as Nguyen et al., 2024) to validate whether the biomechanical advantages of longer InterTan nails translate into improved union and lower failure rates in elderly, comorbid patients.

## Data Availability

The raw data supporting the conclusions of this article will be made available by the authors, without undue reservation.
